# Predicting the distributions of *Scleroderma guani* (Hymenoptera: Bethylidae) under climate change in China

**DOI:** 10.1002/ece3.9410

**Published:** 2022-10-05

**Authors:** Xinqi Deng, Danping Xu, Wenkai Liao, Rulin Wang, Zhihang Zhuo

**Affiliations:** ^1^ College of Life Science China West Normal University Nanchong China; ^2^ Sichuan Provincial Rural Economic Information Center Chengdu China; ^3^ Water‐Saving Agriculture in Southern Hill Area Key Laboratory of Sichuan Province Chengdu China

**Keywords:** climate change, distribution, environmental factors, MaxEnt, *Scleroderma guani*

## Abstract

The wasp *Scleroderma guani* is an important parasitic natural enemy of a variety of stem borers such as longicorn beetles. Studying and clarifying the suitable area of this wasp plays an important role in controlling stem borers. Based on information about the actual distribution of *S. guani* and on a set of environmental variables, MaxEnt niche model and ArcGIS were exploited to predict the potential distribution of this insect in China. This work simulated the geographical distribution of potential climatic suitability of *S. guani* in China at present and in different periods in the future. Combining the relative percent contribution score of environmental factors and the Jackknife test, the dominant environmental variables and their appropriate values restricting the potential geographical distribution of *S. guani* were screened. The results showed that the prediction of the MaxEnt model was highly in line with the actual distribution under current climate conditions, and the simulation accuracy was very high. The distribution of *S. guani* is mainly affected by bio18 (Precipitation of Warmest Quarter), bio11 (Mean Temperature of Coldest Quarter), bio13 (Precipitation of Wettest Month), and bio3 (Isothermality). The suitable habitat of *S. guani* in China is mainly distributed in the Northeast China Plain, North China Plain, middle‐lower Yangtze Plain, and Sichuan Basin, with total suitable area of 547.05 × 10^4^ km^2^, accounting for 56.85% of China’s territory. Furthermore, under the RCP2.6, RCP4.5, and RCP8.5 climate change scenarios in the 2050s and 2090s, the areas of high, medium, and low suitability showed different degrees of change compared to nowadays, exhibiting expansion trend in the future. This work provides theoretical support for related research on pest control and ecological protection.

## Graphical abstract

MaxEnt and ArcGIS can successfully model *Scleroderma guani* distribution. *S. guani* is mainly distributed in the south and the north of China. High suitable habitat of *S. guani* will be increased under climate change. The distribution of *S. guani* is mainly affected by Precipitation of Warmest Quarter and Mean Temperature of Coldest Quarter.

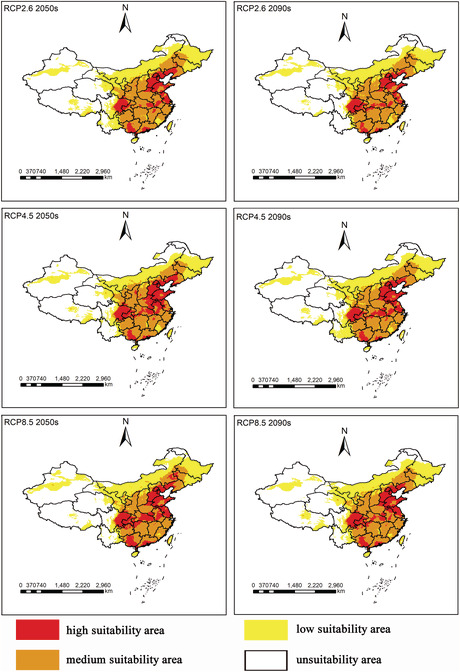

## 1 INTRODUCTION

*Scleroderma guani* (Hymenoptera: Bethylidae), an ectoparasitic wasp, is a natural enemy insect that takes the larvae and pupae of Coleoptera, Lepidoptera, and other insects (particularly long‐horned beetles) as hosts (Zheng et al., 2015). It is widely distributed in China, including Hebei, Shandong, Henan, Guangdong, Hunan, and Jiangsu provinces (Hu et al., 2014). The parasite was first discovered in 1973 and successfully reproduced indoors for the first time in 1977 (Zhang, 2018). The occurrence of *S. guani* is related to the host distribution and is affected by different climate‐related variables, especially temperature and rainfall. It is an effective natural enemy for the prevention and control of borer pests such as long‐horn beetles, buprestid beetles, and some engraver beetles. In nature, if adult wasps locate their hosts and lay eggs smoothly on the surface, this means that parasitism begins. In addition, the insect is an inhibitory parasitic wasp (Luo & Li, 2018; Zhang et al., 2015). Before laying eggs, the female will stab the host, inject the venom to make it anesthetized, and then lay eggs on the host with no resistance (Yao & Yang, 2008). The female *S. guani* also has a stronger ability to hunt and attack hosts. *Scleroderma guani* has a high parasitic rate against small‐ and medium‐sized boring pests (Zhang et al., 2015). Generally, one generation of *S. guani*, involving egg, larva, pupa, and adult, will be completed in about a month at 25°C (Wang et al., 2020). This wasp has a valuable biological control agent.

Ecological Niche Models (ENMs) infer the relationship between species distribution and environmental variables by relating the information on a sample of occurrence data of the target species with the values of the environmental variables on the sample localities and adopt this relationship to estimate the distribution of regions that satisfy the niche requirements of target species (Hutchinson, 1965; Peterson et al., 2011; Zhu et al., 2013), then regarding those areas as parts of the potential distribution. ENMs are crucial tools for ecological research (Booth, 2018). In the past few decades, ENMs have been widely applied to study the distribution of species. Many studies have demonstrated that the MaxEnt model has certain advantages in terms of prediction accuracy, particularly in the case of fewer target species distribution data (Phillips et al., 2006; Saatchi et al., 2008; Yi et al., 2017). Zhang et al. (2016) compared the prediction accuracy of 4 commonly used niche models, and the results showed that MaxEnt model had better prediction effect. Elith et al. (2006) compared the prediction ability of various niche models and concluded that MaxEnt had the highest prediction ability among 16 models. Consequently, MaxEnt was selected as the simulation software in this study. The MaxEnt model has the characteristics of being relatively convenient to use and only requires a small sample size (Ma & Sun, 2018). Since Phillips proposed this model, MaxEnt has been commonly applied in the assessment of potential distribution of species (Zhou et al., 2016), the protection of endangered plant and animal (Zheng et al., 2016), the risk evaluation of species invasion (Rodríguez‐Merino et al., 2018), the assessment of pest and disease spread and control (Zaidi et al., 2016), and good simulation outcomes were obtained. The Maxent model was used to predict the suitable areas of insects under current and future climate conditions, which can clarify the impact of climate change on insect distribution and provide a certain basis for further research on insects (Huang et al., 2020; Zhao & Shi, 2019).

In this work, the MaxEnt and ArcGIS technologies were used to analyze the environmental suitability of *S. guani*, based on known distribution data and combined with environmental data in China. Predicting the current and future potential distribution of *S. guani* will provide theoretical basis for pest control, particularly stem borers.

## 2 MATERIAL AND METHODS

### 2.1 Species data sources and processing

**FIGURE 1:**
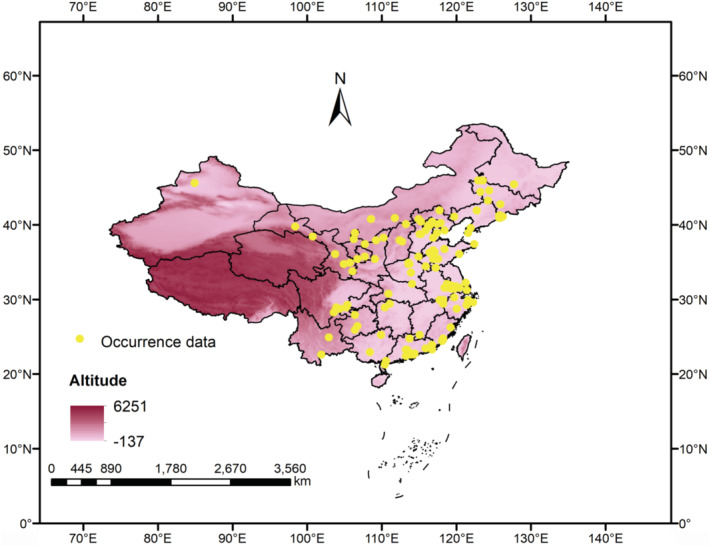
Geographical distribution points of *Scleroderma guani* in China. Yellow points, occurrence data of *S. guani*. Light pink, low altitude. Dark red, high altitude.

The crucial prerequisite for building a niche model is that there should be enough existing species records (Zhang et al., 2019). *Scleroderma guani* first appeared in Guangdong province in 1973 and Shandong province in 1975. Soon afterwards, it was discovered in many provinces of China (Zhang et al., 1987). By querying the records of the Global Biodiversity Information Facility (GBIF, https://www.gbif.org/), consulting published relevant literature and books, and integrating with GPS field survey data, the statistics of the natural distribution points of *S. guani* were obtained. The records were converted to uniform latitude and longitude coordinates (refer to WGS84 geographic coordinates system); the latitude and longitude data of *S. guani* distribution points were confirmed by Google Earth online (http://www.earthol.com/). The collected distribution points of *S. guani* were imported into ArcGIS software. Buffer analysis method was used to screen the obtained distribution points to exclude the influence of over‐fitting simulation caused by large spatial correlation. Since the spatial resolution of environmental variables was 2.5 arc‐min (about 4.5 km^2^), the buffer radius was set to 1.5 km. When the distance between the distribution points is <3 km, only one point was retained. Ultimately, a total of 124 valid sites were obtained (Figure 1); these records were exported as a CSV file for further model analysis.

### 2.2 Environmental factors

The theoretical basis of the ENMs is the concept of ecological niche, which is defined as the position occupied by a population in an ecosystem in time and space and its relationship and role with other populations (Hutchinson, 1965). Environmental variables play an important role in the niche distribution of species (Wang et al., 2019). In order to comprehensively explore the effects of climate on the spread of *S. guani* in China, the environmental variables considered in this work were extracted from the Worldclim database (Version 2.0, http://www.worldclim.org/). Climatic variables used in the model are shown in Table 1, including 19 bioclimate variables and the altitude data selected as topographic factor. The Worldclim bioclimatic variables for the current scenario represented average values for the 1970–2000 period, and the corresponding raster files were downloaded at a spatial resolution of 2.5 arcminutes. The global climate model used in this study was BCC‐CSM1‐1, and it was found that BCC‐CSM1‐1 has a good simulation effect on China's regional climate (Feng, 2012). We obtained future climate data for the 2050s (2041–2060) and 2090s (2081–2100) by the Climate Change, Agriculture and Food Security website (CCAFS, https://ccafs.cgiar.org/). The Fifth Assessment Report of the Intergovernmental Panel on Climate Change (IPCC) considered the four greenhouse gas concentration scenarios (Petersen, 2013). Indeed, Zhang et al. (2014) showed that the RCP4.5 scheme has a higher priority than RCP6.0, so this study did not use the RCP6.0. Three representative concentration pathways (RCPs), comprising the minimum greenhouse gas emission scenario (RCP2.6), the medium greenhouse gas emission scenario (RCP4.5), and the maximum greenhouse gas emission scenario (RCP8.5), were selected to simulate the distribution of the species in this work.

**TABLE 1:** Climatic variables used for predicting potential geographic distribution of *Scleroderma guani*.

Code	Environmental variables
bio1	Annual Mean Temperature
bio2	Mean Diurnal Range (Mean of monthly [max temp–min temp])
bio3	Isothermality (bio 2/bio 7) (*100)
bio4	Temperature Seasonality (SD *100)
bio5	Max Temperature of Warmest Month
bio6	Min Temperature of Coldest Month
bio7	Temperature Annual Range (bio5–bio6)
bio8	Mean Temperature of Wettest Quarter
bio9	Mean Temperature of Driest Quarter
bio10	Mean Temperature of Warmest Quarter
bio11	Mean Temperature of Coldest Quarter
bio12	Annual Precipitation
bio13	Precipitation of Wettest Month
bio14	Precipitation of Driest Month
bio15	Precipitation Seasonality (Coefficient of Variation) 1
bio16	Precipitation of Wettest Quarter
bio17	Precipitation of Driest Quarter
bio18	Precipitation of Warmest Quarter
bio19	Precipitation of Coldest Quarter
Alt	Altitude

### 2.3 MaxEnt modeling

This research aimed at exploring the effects of environmental factors on the distribution of *S. guani*. Combining the determined distribution points with the screened climatic factors, MaxEnt 3.4.4 software was used for simulating the potential distribution of *S. guani*. Among them, 75% of the species occurrence data were randomly selected as the training data set, with the remaining 25% utilized as test data, and the number of repetition trainings was set to 10 to reduce the uncertainty caused by abnormal values in the environmental variables associated with the randomly selected training points. The maximum number of background points was set to 10,000, and bootstrap was selected as replicated run type. During the construction of the initial model, the percent contribution of each environmental variable was detected by Jackknife test of the MaxEnt 3.44 software (Yan et al., 2020), and the variables which had low percent contribution (<1%) were removed (Zhu et al., 2014). After that, the Pearson correlation coefficient (*r*) was analyzed for the remaining climate variables by R software. According to widespread practice in Ecological Niche Modeling, highly correlated variables (|*r*| ≥ 0.8) were removed to exclude the effect of collinearity on model and further improved the veracity of the simulation (Xu et al., 2019). Ultimately, the Maxent model was then refitted using only the highly contributing and uncorrelated environmental predictors.

Then, the distribution territory of *S. guani* in China was extracted by ArcGIS, and the climatic suitability of the wasp was analyzed. The output of the MaxEnt software simulation ranged from 0 to 1, and the value closer to 1 meant the higher the possibility of species presence (Wang et al., 2017). Referring to the IPCC report on the assessment of the possibility of the division of the method (IPCC, 2007), combined with the actual situation of *S. guani*, the habitat suitability was divided into four levels and indicated in different colors in this work: high suitability area (0.66–1, red), medium suitability area (0.33–0.66, orange), low suitability area (0.05–0.33, yellow), and unsuitability area (0–0.05, white).

### 2.4 Model optimization and evaluation

In this research, the default parameters of the MaxEnt software were RM = 1, FC = LQHPT. In R software, two parameters of regularization multiplier (RM) and feature classes (FC) were adjusted by calling *ENMeval* package to optimize MaxEnt model (Kass et al., 2021; Zhou et al., 2016). The MaxEnt model provided five features, which were L(linear), Q(quadratic), H(hinge), P(product), and T(threshold), and they can generate 31 different combinations. The RM parameter was set from 0.1 to 4, and the interval was 0.1, so that 40 RM values were evaluated. The *ENMeval* packet was used to test the above 1240 parameter combinations. AUC_DIFF_ (the difference between training set AUC and test set AUC) and test omission rate were used to test the fit of the model to species distribution. The closer the test omission rate to the theoretical omission rate, the higher the accuracy of the constructed model is (Shcheglovitova & Anderson, 2013). Akaike information criterion (AIC) was used to evaluate the fitting degree and complexity of different parameter combinations. The parameter combinations leading to the lowest AICc value (ΔAICc = 0) were selected to fit the optimized model (Jia et al., 2019).

After the optimization, the optimal parameters were used to simulate and predict the suitable habitat of *S. guani* in different periods. The accuracy of the simulation results was evaluated using the receiver operating characteristic curve (ROC), and the area under the curve (AUC) was used to evaluate the predictive performance of the model (Na et al., 2018). The value of AUC changes between 0 and 1, and the value of AUC is <0.8 means the simulation result has low reliability, and the AUC value is in the range of 0.8–0.9, which means the simulation result is more accurate. The AUC value is in the range of 0.9–1, which means the simulation result is very accurate (Walden‐Schreiner et al., 2017). In addition, since TSS and Kappa are not affected by the size of the verification set (Allouche et al., 2006), we also chose TSS and Kappa to evaluate the accuracy of the model. The Kappa value above 0.75 indicates that the performance of this model is excellent. The TSS value ranges from −1 to 1. The closer the TSS value is to 1, the better the accuracy of the model, and the closer to −1 the lower the accuracy (Yan et al., 2020). TSS = 0 indicates that the model does not recognize omission and commission errors (Li et al., 2021).

## 3 RESULTS

### 3.1 Model optimization results and accuracy evaluation

The MaxEnt model was optimized with the *ENMeval* R package, and the current and future potential distribution areas of *S. guani* were predicted. When FC = LQP and RM = 0.2, the delta AIC value reached its minimum (ΔAICc = 0), indicating that the model under this parameter combination was the optimal one. The optimized AUC_DIFF_ value, mean AUC value, mean TSS, mean Kappa were 0.015, 0.988, 0.852, and 0.801, respectively. The results indicated that the optimized MaxEnt model could accurately simulate the potential geographical distribution of *S. guani*.

### 3.2 Model performance and Key environment variables

Combined with the percent contribution and Pearson’s correlation coefficient, the eight key environmental variables were screened out, and the species distribution model was reconstructed. Table 2 displayed the percent contribution and permutation importance of eight environmental variables which affected the distribution of *S. guani*: precipitation of warmest quarter (bio18), temperature seasonality (bio4), precipitation seasonality (bio15), precipitation of wettest month (bio13), mean temperature of coldest quarter (bio11), isothermality (bio3), altitude (alt), and precipitation of coldest quarter (bio19). The percent contribution of bio18 was the highest, reaching 42.1%, followed by bio4 (17.3%) and bio15(10.5%). The accumulated percent contribution of those key environmental variables accounted for 89.5%, indicating that the above‐mentioned eight environmental variables contained effective information on the most suitable habitat of *S. guani* and were the key to simulating the potential geographic distribution of *S. guani*.

**TABLE 2:** Percent contribution and the permutation importance of environmental variables affecting the distribution of *Scleroderma guani.*

Code	Environmental variables	Percent contribution/%	Permutation importance/%
bio18	Precipitation of Warmest Quarter	42.1	18
bio4	Temperature Seasonality (SD *100)	17.3	5.5
bio15	Precipitation Seasonality (Coefficient of Variation) 1	10.5	2.6
bio13	Precipitation of Wettest Month	5.1	0.4
bio11	Mean Temperature of Coldest Quarter	4.5	22
bio3	Isothermality (bio2/bio7) (*100)	4.4	2.9
Alt	Elevation	3.4	1.1
bio19	Precipitation of Coldest Quarter	2.2	0.4

**FIGURE 2:**
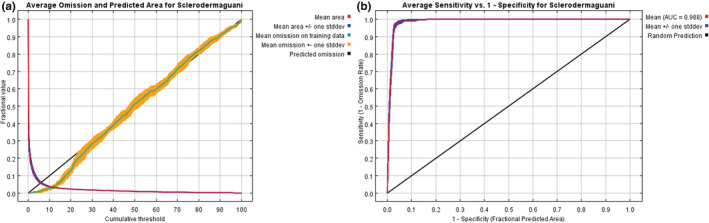
Predictive effect of the MaxEnt model of *Scleroderma guani*. (a) Curve of omission and predicted area. (b) ROC curve of potential distribution prediction.

The Pearson correlation coefficients of the above‐mentioned eight environmental variables are shown in Table 3. The results illustrated that except for |*r*| = 0.851 of both bio3 and bio4, these values were lower than 0.8. As the percent contribution of bio3 and bio4 in this study were relatively large, and these two factors have a great impact on the distribution of many insects (Wang et al., 2017; Xu, Liu, et al., 2021; Xu, Tang, et al., 2021; Zhao & Shi, 2019), so they are retained. As shown in Figure 2a, the prediction omission rate showed a high agreement with the test sample omission rate, which indicated the good prediction effect of the model. Figure 2b showed the ROC curve of the model and exhibited that the AUC value reached 0.988, which indicated the model's prediction accuracy was excellent. This model was reliable for confirming the potential distribution of *S. guani* in China.

**TABLE 3:** Pearson’s correlation coefficients of crucial environmental factors.

	bio3	bio4	bio11	bio13	bio15	bio18	bio19
bio4	−0.851						
bio11	0.797	−0.703					
bio13	0.631	−0.563	0.594				
bio15	−0.053	0.032	−0.334	−0.107			
bio18	0.482	−0.366	0.526	0.785	−0.303		
bio19	0.479	−0.435	0.377	0.628	−0.299	0.432	
alt	−0.231	0.123	−0.622	−0.319	0.566	−0.357	−0.233

### 3.3 Predicting the current distribution of *S. guani* in China

**FIGURE 3:**
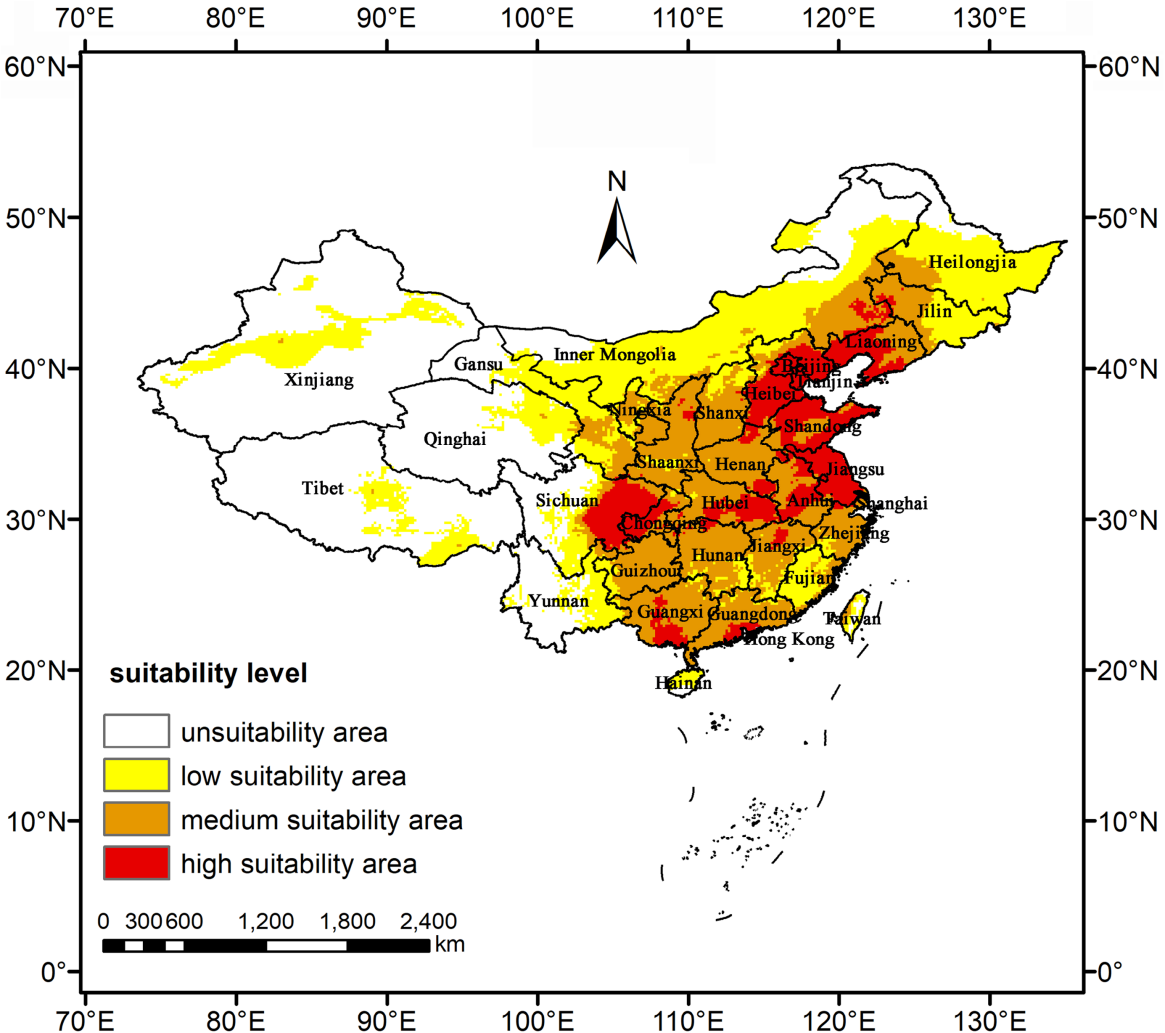
Current suitable distribution of *Scleroderma guani* in China. Red, high suitability area with the probability of .66–1. Orange, medium suitability area with the probability of .33–.66. Yellow, low suitability area with the probability of .05–.33. White, unsuitability areas with the probability of 0–.05.

Projection of the suitability for *S. guani* across China, according to the optimized Maxent model, is shown in Figure 3. The statistics for the predicted areas of *S. guani* in different provinces are displayed in Table 4. The results displayed that the contemporary distribution of high suitability area was distributed in subtropical and warm temperate regions, mainly concentrated in low‐altitude and basin areas, especially the Northeast China Plain, North China Plain, middle‐lower Yangtze Plain, and Sichuan Basin. The total suitable area was 547.05 × 10^4^ km^2^, accounting for 56.85% of China's total land area. The provinces with large suitable areas included Beijing (96.88%), Jiangsu (96.05%), Shandong (74.77%), Hebei (66.43%), and Liaoning (58.87%). In addition, Tianjin (100%) and Hong Kong (100%) were all listed as highly suitable areas. The distribution of moderately suitable area was closely connected with the high suitable area, and the distribution area was wide. With the change of time, there is a great possibility to transform to high suitable area.

**TABLE 4:** Predicted suitability for *Scleroderma guani* in China under current climatic conditions.

Province	High suitability		Medium suitability		Low suitability		No suitability	
	Predicted area (×10^4^ km^2^)	Area ratio (%)^a^	Predicted area (×10^4^ km^2^)	Area ratio (%)^a^	Predicted area (×10^4^ km^2^)	Area ratio (%)^a^	Predicted area (×10^4^ km^2^)	Area ratio (%)^a^
Hebei	13.03	66.43	3.89	19.83	2.70	13.74	0.00	0.00
Sichuan	12.00	26.42	4.39	9.66	9.64	21.22	19.40	42.69
Shandong	11.53	74.77	3.89	25.23	0.00	0.00	0.00	0.00
Jiangsu	9.45	96.05	0.39	3.95	0.00	0.00	0.00	0.00
Liaoning	9.23	58.87	6.45	41.13	0.00	0.00	0.00	0.00
Hubei	6.22	35.22	11.25	63.68	0.19	1.10	0.00	0.00
Anhui	5.56	41.58	7.81	58.42	0.00	0.00	0.00	0.00
Chongqing	4.03	52.35	3.42	44.40	0.25	3.25	0.00	0.00
Guangxi	4.03	19.13	16.12	76.52	0.92	4.35	0.00	0.00
Henan	2.95	18.09	13.31	81.74	0.03	0.17	0.00	0.00
Inner Mongolia	2.64	2.04	17.92	13.86	75.70	58.52	33.10	25.59
Guangdong	1.89	12.08	12.78	81.71	0.97	6.22	0.00	0.00
Beijing	1.72	96.88	0.06	3.13	0.00	0.00	0.00	0.00
Tianjin	1.22	100.00	0.00	0.00	0.00	0.00	0.00	0.00
Jiangxi	1.00	6.57	11.28	74.09	2.95	19.34	0.00	0.00
Hunan	0.83	4.30	15.81	81.64	2.72	14.06	0.00	0.00
Jilin	0.78	3.65	9.56	44.85	10.98	51.50	0.00	0.00
Shaanxi	0.72	3.51	18.31	89.05	1.53	7.43	0.00	0.00
Zhejiang	0.31	3.28	7.98	85.67	1.03	11.04	0.00	0.00
Shanxi	0.19	1.22	12.25	76.83	3.50	21.95	0.00	0.00
Shanghai	0.17	27.27	0.44	72.73	0.00	0.00	0.00	0.00
Hong Kong	0.14	100.00	0.00	0.00	0.00	0.00	0.00	0.00
Guizhou	0.14	0.88	13.56	85.46	2.17	13.66	0.00	0.00
Yunnan	0.11	0.33	1.36	3.98	10.81	31.63	21.90	64.07
Gansu	0.06	0.13	10.00	24.11	16.31	39.32	15.12	36.44
Fujian	0.00	0.00	2.95	26.77	8.06	73.23	0.00	0.00
Taiwan	0.00	0.00	0.67	20.69	1.58	49.14	0.97	30.17
Hainan	0.00	0.00	0.00	0.00	2.20	72.48	0.83	27.52
Heilongjiang	0.00	0.00	6.11	11.23	34.71	63.76	13.62	25.01
Xinjiang	0.00	0.00	0.08	0.05	25.48	14.53	149.78	85.42
Ningxia	0.00	0.00	2.97	57.84	2.17	42.16	0.00	0.00
Qinghai	0.00	0.00	0.58	0.82	11.25	15.73	59.69	83.45
Xizang	0.00	0.00	0.11	0.10	13.53	11.83	100.76	88.07
Total area	89.95		215.73		241.37		415.17	

^a^ The ratio of the predicted area to the total predicted land area of the corresponding province.

### 3.4 Potential distribution of *S. guani* in future period

**FIGURE 4:**
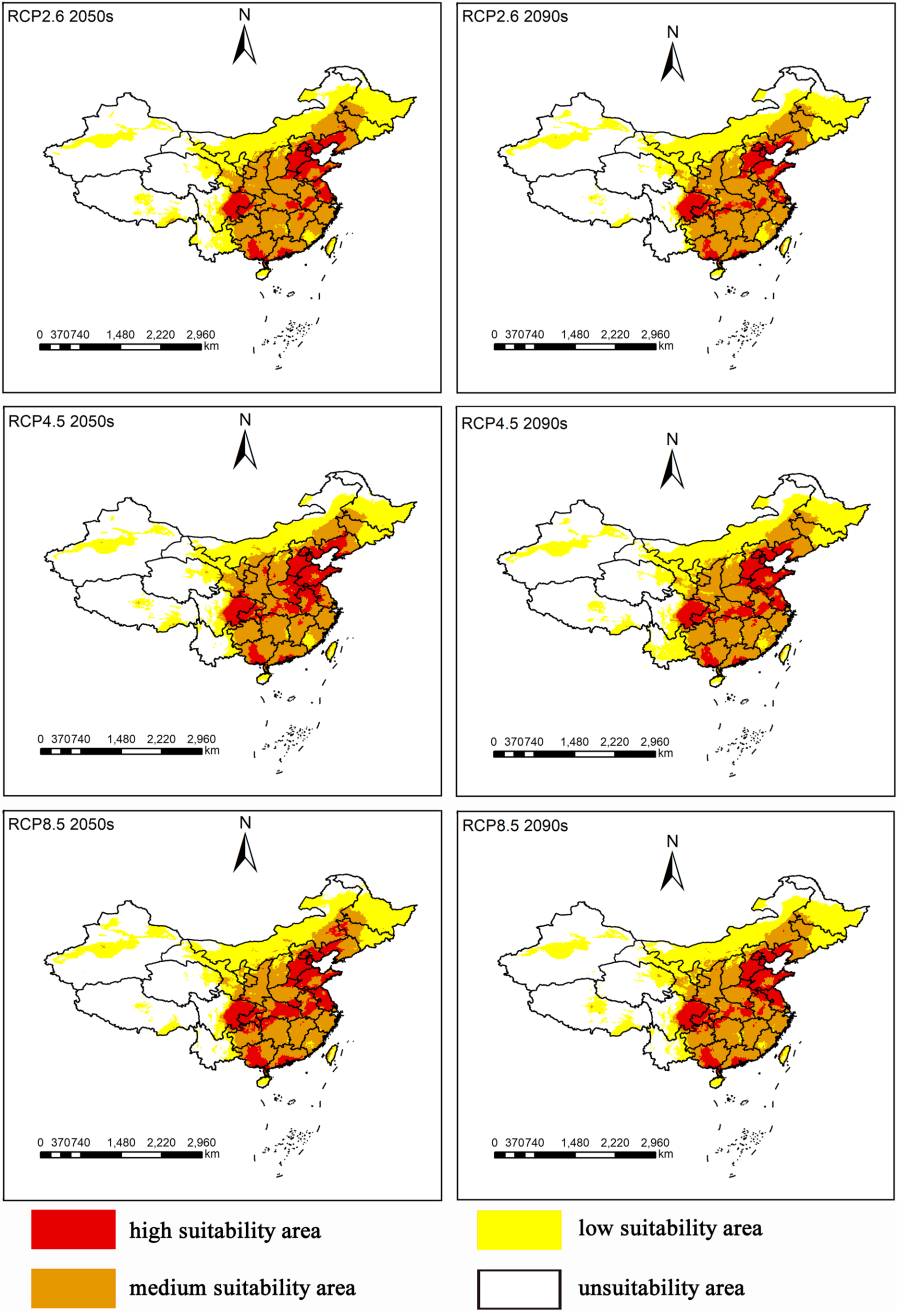
Potential distribution of *Scleroderma guani* in future period (2050s, 2090s) under the RCP2.6, RCP4.5 and RCP8.5 climate change scenarios. Red, high suitability area with the probability of .66–1. Orange, medium suitability area with the probability of .33–.66. Yellow, low suitability area with the probability of .05–.33. White, unsuitability areas with the probability of 0–.05.

Habitats predicted by the Maxent model as suitable for *S. guani* in China under the RCP2.6, RCP4.5, and RCP8.5 climate change scenarios are shown in Figure 4. Compared with the predicted results under the current climate, there was an obvious change in tendency of the predicted high, medium, and low suitable area in the 2050s and 2090s. The suitable distribution area showed a trend of northwest expansion.

**FIGURE 5:**
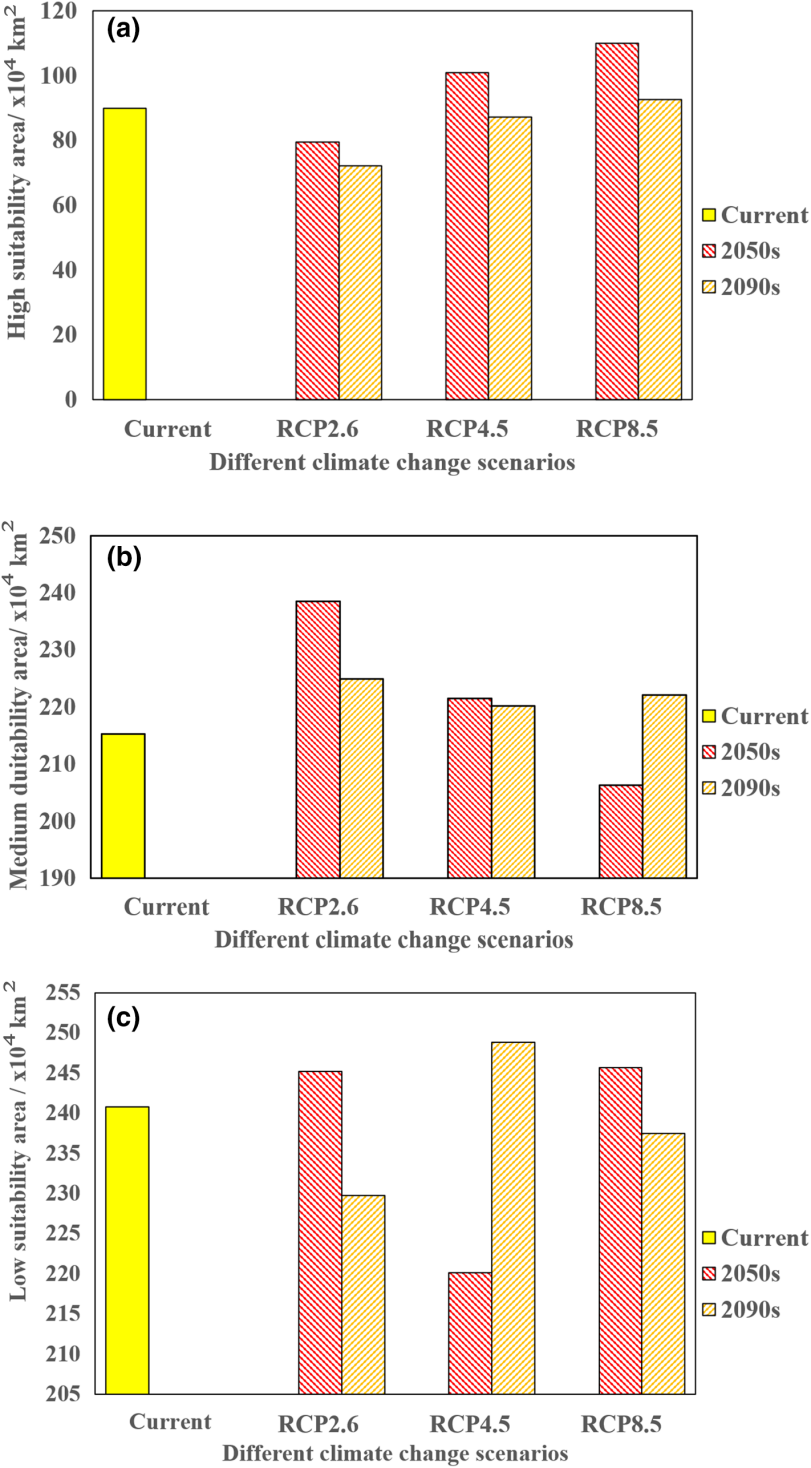
The area change of suitable habitat for *Scleroderma guani* under different climate change scenarios. (a) area of high suitability. (b) area of medium suitability. (c) area of low suitability.

As shown in Table 5 and Figure 5, under the RCP2.6 scenario, model projections suggest that by the 2090s, the high suitability area will decrease the most compared to current conditions, and the reduced areas will be 17.64 × 10^4^ km^2^, accounting for 19.65% of the current predicted one. From now to the 2050s and the 2090s, there will be a trend of transformation from high suitable area to medium suitable. Among them, the highly suitable areas will have significantly declined in the provinces of Hubei, Anhui, Jiangsu, and Liaoning. Under the RCP4.5 scenario, the extent will convert low suitable areas to medium and high suitable in the 2050s. The extent of high suitable area will rise by 11.06 × 10^4^ km^2^ in 2050s and fall by 2.58 × 10^4^ km^2^ in 2090s, accounting for 12.33% and 2.87% of the current predicted one, respectively. From present to the 2050s and to the 2090s, the moderate suitable area increased in extent, with the percentage rising by 2.91% and 2.45%, respectively. Many currently low suitability areas in the provinces of Fujian, Zhejiang, Jiangxi, and Hunan will turn to medium suitability. Under the RCP8.5 scenario, the high suitable areas will increase by 20.15 × 10^4^ km^2^ and 2.74 × 10^4^ km^2^ from current to the 2050s and the 2090s, respectively. They account for 22.46% and 3.06% of the current, respectively. The significantly increased high suitability areas will mainly be distributed in Guangxi, Guizhou, and Hunan provinces.

**TABLE 5:** Predicted suitable areas for *Scleroderma guani* under current and future climatic conditions.

Decade	Scenarios	Predicted area (×10^4^ km^2^)			Comparison with current distribution (%)			Total change (×10^4^ km^2^)
		Low suitability	Medium suitability	High suitability	Low suitability	Medium suitability	High suitability	
Current		240.82	215.23	89.75				
2050s	RCP2.6	245.17	238.52	79.35	1.81	10.82	−11.58	17.24
	RCP4.5	220.14	221.49	100.81	−8.59	2.91	12.33	−3.35
	RCP8.5	245.64	206.27	109.90	2.00	−4.16	22.46	16.02
2090s	RCP2.6	229.78	224.90	72.11	−4.58	4.50	−19.65	−18.99
	RCP4.5	248.86	220.50	87.17	3.34	2.45	−2.87	10.73
	RCP8.5	237.44	222.08	92.49	−1.40	3.18	3.06	6.21

### 3.5 Relationship between environmental variables and geographical distribution

**FIGURE 6:**
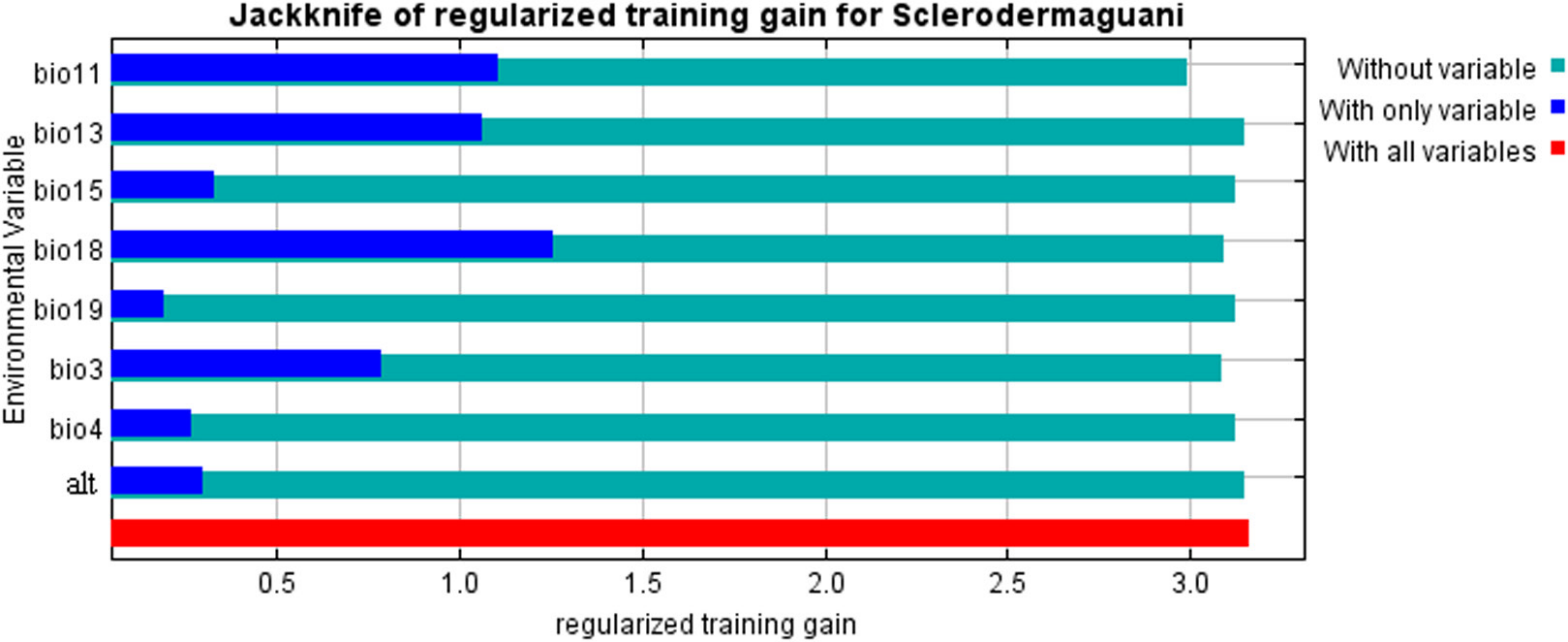
Importance of environmental variables to *Scleroderma guani* by Jackknife test.

**FIGURE 7:**
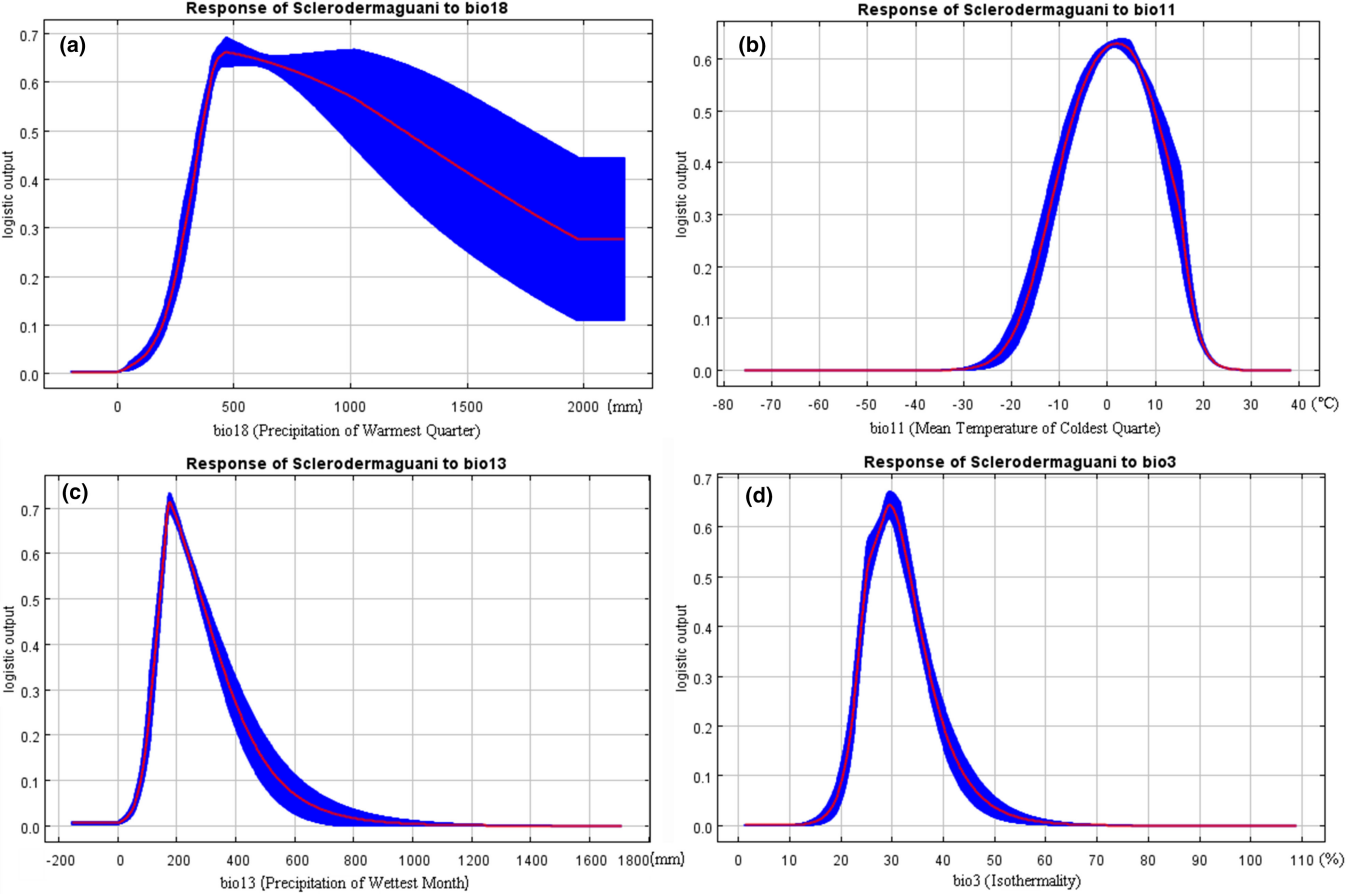
Response curves between environmental variables and predicted suitability. a–d were bio18, bio11, bio13, and bio3, respectively.

The selected environmental variables that have a noticeable impact on the distribution of *S. guani* were analyzed by Jackknife test method. As shown in Figure 6, all the predictors affected the potential distribution of *S. guani* to some extent, with bio18 being the most important when used alone. The blue band represented the importance of the variable to species distribution in Figure 6. The longer the band, the more important the variable was to species distribution. Figure 7 showed how predicted suitability varied with increasing values of the selected variables. Referring to the grading method of the IPCC (IPCC, 2007), the range of environmental variables suitable for *S. guani* distribution was divided, using 0.33 as a threshold. The results revealed that in the appropriate range, when the environmental variable value was below the optimal value, the distribution probability increased with the environmental variable, when the value was greater than the optimal value, it decreased as the environmental variables increased.

According to the response curve of environmental variables to distribution probability in the MaxEnt model (Figure 7), the appropriate range of environmental variables to the potential distribution of *S. guani* was determined, as shown in Table 6. The appropriate value range of precipitation of warmest quarter (bio18) was 302.3–1784.2 mm, and the optimal value was 460.7 mm. This relatively wide range of values for bio18 suggests that *S. guani* can occur under simultaneously warm and humid conditions. At 302.3–460.7 mm, the predicted suitability of *S. guani* increased rapidly with the raising of precipitation of warmest quarter and decreased slowly with the increase of precipitation of warmest quarter at 460.7–1784.2 mm. When the mean temperature of coldest quarter (bio11) was −11.5°C–14.9°C, the predicted suitability of *S. guani* was higher than 0.33, and the predicted suitability was the highest at 2°C, reaching 0.63. The small range of appropriate values for bio11 suggested that *S. guani* is highly sensitive to extreme temperature changes. When the precipitation of wettest month (bio13) was lower than 120.0 mm, the suitability of predicted *S. guani* was lower than 0.33. With the increase in precipitation, the suitability of prediction increased quickly and reached the peak at 175.1 mm. When the precipitation exceeded 364.5 mm, the suitability of predicted dropped again below 0.33. A slight change in bio3 can have a significant effect on the distribution of *S. guani*, suggesting that it preferred areas with less temperature variation. The appropriate range of the response curve for isothermality (bio3) was 23.4%–36.9%, and the most appropriate value was 29.6%. The suitable values range of bio15, alt, bio4, and bio19 are all shown in Table 6.

**TABLE 6:** Suitable range of environmental variables for potential distribution of *Scleroderma guani.*

Environmental variables	Suitable range	Optimum value
Bio18/mm	302.3–1784.2	460.7
Bio11/°C	−11.5–14.9	2.00
Bio13/mm	120.0–364.5	175.1
Bio3/%	23.4–36.9	29.6
Bio15/mm	48.2–146.1	106.7
Alt/m	−52.3–1602.7	15.6
Bio4/°C	4.5–13.9	10.4
Bio19/mm	1.3–298.8	134.6

## 4 DISCUSSION

The MaxEnt model was used to simulate the potential geographical distribution of *S. guani* in China, and the results showed that the highly suitable areas were mainly located in Sichuan, Hebei, Shandong, Jiangsu, Guangdong, Beijing, Chongqing, and other regions, which was consistent with relevant previous research on this species (Xiao & Wu, 1983; Zheng et al., 2015), and the predicted suitable distribution ranges were broader in this study. MaxEnt quantifies the distribution of species based on the maximum entropy theory. This method unrestricted unknown distribution information, and compared with other similar models, MaxEnt has more advantages and has been widely used in China and abroad (Wang et al., 2021). The MaxEnt model was evaluated by using Kappa, TSS, ROC curves, and the AUC values in this study. The results indicated that the model has a particularly good prediction effect on the distribution area of *S. guani* and has a very high reliability.

In this research, the most important environmental variables limiting *S. guani* distribution, which included bio18, bio11, bio13, and bio3, were screened using Jackknife test combined with Pearson’s correlation coefficient, and the result indicated that precipitation and temperature jointly constrained the current distribution pattern of *S. guani. Scleroderma guani* has a relatively high tolerance to humidity and can develop normally under the condition of relative humidity of 40%–90% (Yao et al., 1983). The range of bio18 from 302.34 mm to 1784.19 mm was suitable for *S. guani* occurrence in this research, and the ranges of bio13 and bio19 were also relatively wide, which was consistent with the results of predecessors (Yao et al., 1983). Wang et al. (2004) discovered that the parasitism rate of *S. guani* is inversely related to temperature. Li et al. (1984) revealed that the reproductive cycle and survival rate of *S. guani* were markedly diverse under different temperature and humidity. Temperature and humidity are closely related to the growth and development of *S. guani* (Zhao, 2019). The female wasps are not able to lay eggs at 15°C. It takes 53–62 days to complete a generation at 23.1°C, 29–30 days at 25.9°C, 21–24 days at 28–30°C (Yao et al., 1983). The starting temperatures of egg, larva, and pupa are 60.18, 169.71, and 219.00 day degrees, respectively (Yao et al., 1983). The temperature range of artificial reproduction in the room of *S. guani* is 22–28°C, the optimum temperature is 26°C, the relative humidity is 60%–80%; in this temperature and humidity interval, the vaccination success rate and spawned volume of *S. guani* are higher (Zhou et al., 2005). All the above results indicated that temperature and precipitation play a key role in *S. guani*. This study showed that the suitable distribution range of bio11 was −11.5°C–14.9°C, and the altitude above 1602.7 m will not be suitable for the distribution of *S. guani*. Studies have found that the adults and pupae of *S. guani* can withstand the low temperature of −24°C and can overwinter in the area at an altitude of 1200–1450 m, but above 1700 m, *S. guani* cannot survive the winter due to the low temperature (Chen & Cheng, 2000). This is consistent with the results of this research.

*Scleroderma guani* ecological suitability distribution map based on the MaxEnt model showed that the high suitability areas were predominantly distributed in the southern and northern regions of China, mainly in the Northeast China Plain, the North China Plain, the Sichuan Basin, and the middle‐lower Yangtze Plain. The climate of these regions respectively belongs to temperate monsoon climate, subtropical monsoon climate, and tropical monsoon climate, and the annual rainfall is 400–3000 mm, and the annual accumulated temperature is over 3000°C. They are characterized by high temperatures and rainfall in summer, wet and hot periods, cold and dry winter, and four distinct seasons.

The temperature and humidity in these areas are in line with the living habits of *S. guani*. The unsuitable distribution areas of *S. guani* are predominantly distributed in northwest China and the Qinghai‐Tibet region, mainly including the Qinghai‐Tibet Plateau, Xinjiang, Gansu, and Qinghai. It may be that the climate in these regions has the characteristics of strong solar radiation, large temperature difference between day and night and low temperature (Liu et al., 2013), which is not conducive to survival of *S. guani*. Under different RCP combinations, the scope of the total suitable area of *S. guani* showed an overall expansion trend in the 2050s and 2090s, and the incremental change was not particularly evident, which may be related to the wide ecological range of *S. guani*, which could adapt to a variety of external environments. Under the high emission scenario (RCP 8.5), the high suitable area of *S. guani* will have an obvious expansion trend, with an increase range of 3.06%–22.46% (Table 5). In the future, the suitable range in Qinghai Tibet region will decline, while that in the northwest of China will increase. Climate warming is driving the expansion of *S. guani* suitable habitat, and it is anticipated that the suitable habitat will shift to higher altitude and higher latitude in the future.

**FIGURE 8:**
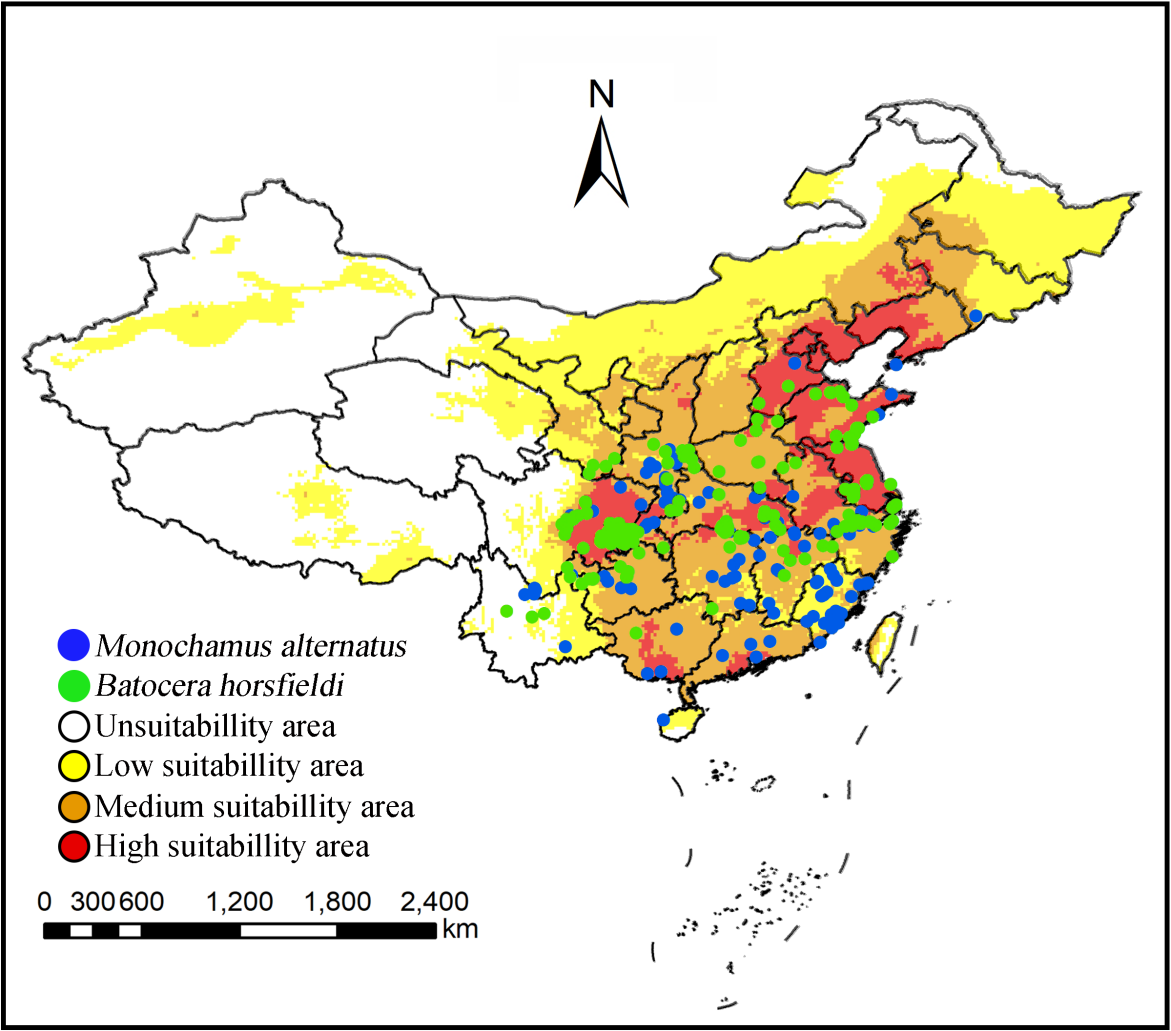
Actual distribution of two host species of *Scleroderma guani*, *Monochamus alternatus* (blue dots) and *Batocera horsfieldi* (green dots), overlaid on the map showing predicted suitability for *S. guani* under current conditions.

As the dominant natural enemy of stem borers, *S. guani* has been widely used in biological control (Yang et al., 2014; Zhang et al., 2021). Large‐scale and low‐cost breeding of *S. guani* has become one of the research hot topics in the field of biological control applications (Liu et al., 2011). The suitable distribution area of *S. guani* was relatively extensive, and the distribution of the host will affect its distribution. There are many kinds of hosts for the wasp, but *Monochamus alternatus* and *Batocera horsfieldi*, which have caused great economic losses, are its main hosts in China (Chen et al., 2008; Zhou et al., 2020). The distribution of hosts was obtained by surveying relevant literature, books, and the GBIF website, and it is shown in Figure 8. The blue dots represented *M. alternatus*, and the green represented *B. horsfieldi*. The host distribution area was included in the current predicted potential distribution area of *S. guani*. According to the research, *M. alternatus* is mainly distributed in Shandong, Henan, Anhui, Guangxi, and Guizhou (Xu et al., 2020), and *B. horsfieldi* mainly distributed in Jiangsu, Hebei, Anhui, Hubei, and Sichuan (Shi et al., 2013). These results indicated that the environmental conditions suitable to the two above‐mentioned hosts and to *S. guani* extensively overlap, and it was viable to utilize *S. guani* for biological control.

The ENMs only describe the basic ecological requirements of species, not the actual ecological requirements. When ENMs are conducted to predict the potential distribution of species, a variety of biological and non‐biological factors affecting the distribution of species are easily overlooked (Xu, Liu, et al., 2021; Xu, Tang, et al., 2021). Up to now, ROC curve analysis has been widely adopted in the evaluation of discrimination performance when modeling the potential distribution of species, and it reveals the performance of the MaxEnt model (Zhang et al., 2021). The MaxEnt model has general advantages in predicting the potential distribution of species, but still has some limitations (Xu et al., 2019). The feedback curve only shows the influence of a single environmental factor, ignoring the interaction between variables. It is impractical to consider all environmental factors comprehensively in a particular model analysis, so it may be more efficient to treat the model as a base niche model (Chakraborty et al., 2016). Distribution and modeling results are also influenced by other intrinsic factors (distance and rate of dispersal of species and time of formation) and extrinsic factors (human activities and natural enemies) (Gao et al., 2021). The work employed limited occurrence data and considered only the environmental factors associated with temperature and rainfall and did not take into account the influence of biological factors such as host distribution and diffusion, predators, and other environmental factors such as human interference, which may have an impact on the accuracy of the prediction results. Hereafter, biological and non‐biological factors such as human activities and host types can be incorporated into the model when studying the suitable area of *S. guani* in order to improve the accuracy of model predictions.

## 5 CONCLUSIONS

This research applied MaxEnt model and ArcGIS technology to successfully calculate the current and future suitable habitat distribution of *S. guani* in China, according to the known distribution information and climate factors. The results revealed that the suitable areas were distributed in low‐altitude areas, and the high suitable areas were mainly concentrated in the coastal area of northeast plain, North China plain and Sichuan Basin. The vital environmental variables that impacted the distribution were precipitation of warmest quarter (bio18), mean temperature of coldest quarter (bio11), precipitation of wettest month (bio13), and isothermality (bio3). The distribution range of *S. guani* in high suitable areas showed a trend of further expansion. This study will provide reference for expanding current knowledge about the environmental drivers of *S. guani* distribution, so as to facilitate its use as biological control agent against stem borers and other pest species. This study explored the impact of the Maxent model on the ecological distribution of *S. guani*, and further research will be carried out in the future combining more ENM and more environmental factors.

## AUTHOR CONTRIBUTIONS

**Xinqi Deng:** Conceptualization (equal); data curation (supporting); formal analysis (equal); software (supporting); writing – original draft (lead). **Danping Xu:** Conceptualization (equal); data curation (supporting); funding acquisition (equal); software (supporting); writing – review and editing (supporting). **Wenkai Liao:** Data curation (lead); investigation (equal); software (supporting); writing – original draft (supporting). **Rulin Wang:** Funding acquisition (equal); methodology (equal); resources (supporting); software (lead); visualization (equal). **Zhihang Zhuo:** Conceptualization (equal); funding acquisition (equal); project administration (lead); resources (lead); software (lead); supervision (lead); validation (lead); writing – review and editing (lead).

## CONFLICT OF INTEREST

None.

## Data Availability

The data supporting the results are available in a public repository at: https://doi.org/10.6084/m9.figshare.19344893.v2.
